# Supplementary figures and images for: The syntenic long non-coding RNA DANCR is an essential regulator of zebrafish development and a human melanoma oncogene
Source: PLoS Genet. 2025 Dec 3;21(12):e1011970. doi: 10.1371/journal.pgen.1011970 (PMC12694859; doi:10.1371/journal.pgen.1011970)

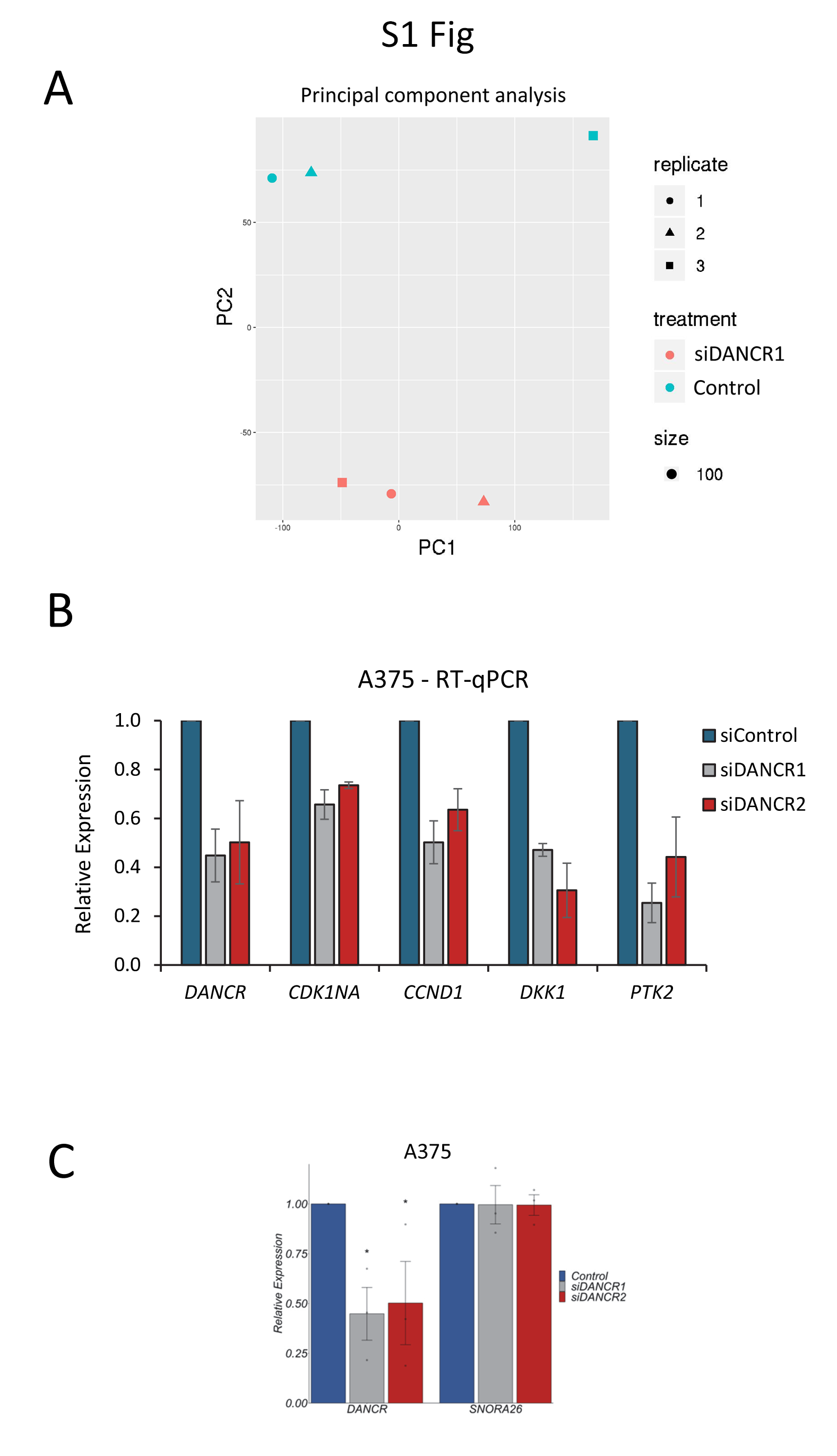

Supplement: S1 Fig — (A) Principal component analysis (PCA) shows that control and siDANCR knockdown samples cluster separately. (B, C) DANCR was depleted in A375 cells using two independent siRNAs. Three days later DANCR and (B) the expression of the indicated DANCR target genes or (C) SNORA26 levels were determined using RT-qPCR. POLII was used as a reference gene. Results are presented as mean + /- SEM., n = 3. Two-tailed two sample t-test p < 0.05. Individual dots represent separate biological replicates. (TIF) [file pgen.1011970.s001.tif]

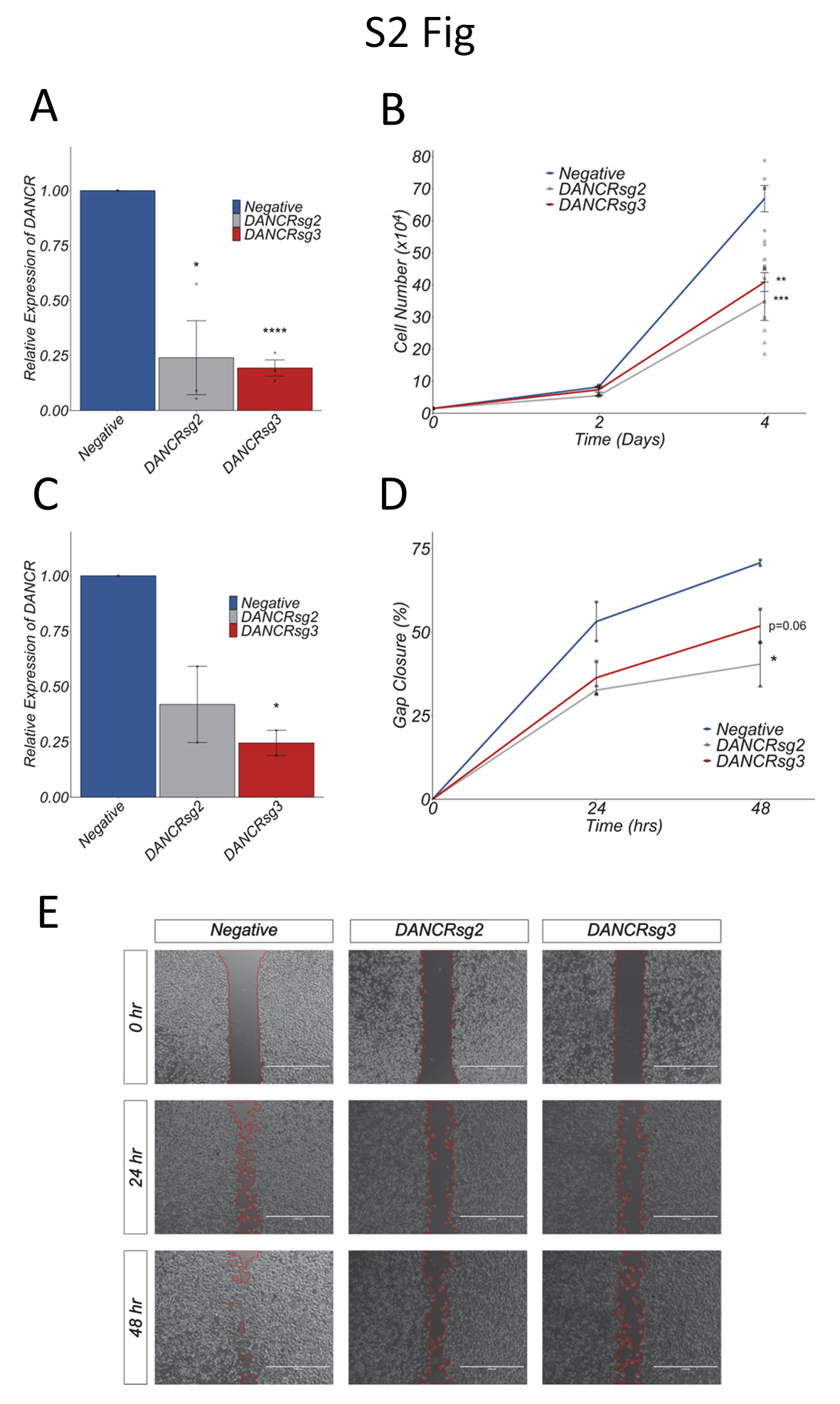

Supplement: S2 Fig — DANCR expression was silenced in A375 cells by dCas9-KRAB mediated CRISPRi using two independent sgRNAs targeting the DANCR promoter. Three days later DANCR levels were measured using RT-qPCR and proliferation (A, B) or wound healing (C, D, E) assays set up. Expression changes are shown relative to a non-targeting control sgRNA (set at 1). POLII was used as a reference gene. For proliferation analysis, cells were seeded in a 6-well plate and the total number of cells were counted at days 0, 2 and 4 (B). For wound healing assays, cells were first treated with mitomycin-C to block cell proliferation and migration was then determined using Ibidi chambers (Culture-Inserts 2 Well). The gap was imaged at 0, 24 and 48 hours and percentage gap closure calculated using the ImageJ Wound Healing plugin (D, E). Statistical analysis was performed at the 48-hour time point. All results presented as mean + /- SEM., n ≥ 2. Two-tailed two sample t-test p < 0.05*, p < 0.01**, p < 0.001***, p < 0.0001****. Individual dots represent separate biological replicates. (TIF) [file pgen.1011970.s002.tif]

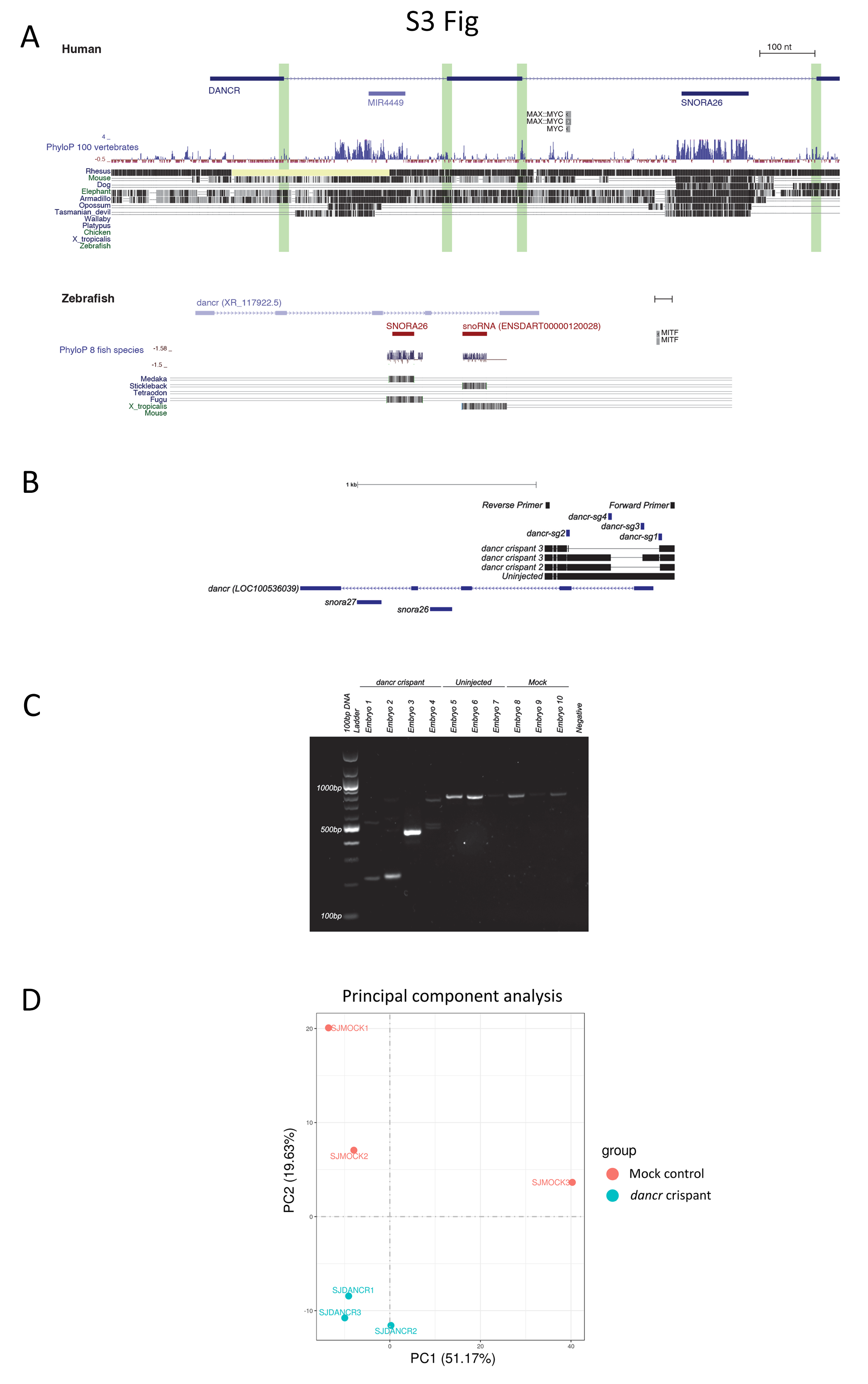

Supplement: S3 Fig — (A) Genome browser view of the human DANCR locus with the 5’ end of a representative isoform and alignment of 100 vertebrate species. Splice sites with positive PhyloP scores are shaded and a predicted MYC binding site from the JASPAR database is shown. Bottom: Genome browser view of zebrafish dancr as annotated in RefSeq displaying the snoRNA positions from Ensembl, MITF binding site motif and alignment of 8 fish species. (B) Genome browser view displaying the zebrafish dancr locus (GRCz10/danRer10). The location of the sgRNAs used to guide CRISPR/Cas9 mediated deletion of the dancr promoter and PCR primers flanking the targeted deletion site for screening are shown. Alignment of representative PCR product sequences indicate the position of the dancr promoter deletions. (C) PCR amplification of the genomic region flanking the proposed dancr promoter deletion was performed using genomic DNA extracted from individual dancr crispant, uninjected and mock control embryos at 24 hpf. A negative control containing water instead of genomic DNA was also used. PCR products were analysed by agarose gel electrophoresis. An expected band of 725 bp corresponding to wild type sequence was amplified in the uninjected and mock control embryos. Multiple bands of varying sizes, due to the mosaic deletions caused by multiple sgRNAs targeting the dancr promoter, were generated in the F0 dancr crispant embryos. (D) PCA analysis on the gene expression value (FPKM) separates the control and knockdown samples. (TIF) [file pgen.1011970.s003.tif]
