# Supplementary material for: The syntenic long non-coding RNA DANCR is an essential regulator of zebrafish development and a human melanoma oncogene
Source: PLoS Genet. 2025 Dec 3;21(12):e1011970. doi: 10.1371/journal.pgen.1011970 (PMC12694859; doi:10.1371/journal.pgen.1011970)
Supplement: Attachment — Submitted filename: Jones et al point by point reviewers response.docx [file pgen.1011970.s010.docx]

**Reviewers' comments:**

Reviewer's Responses to Questions

Reviewer #1: The paper entitled “The syntenic long non-coding RNA DANCR is an essential regulator of zebrafish development and a human melanoma oncogene” adds an important set of data to the misconception that lincRNAs are solely fine-tuners or not important at all for the organismal homeostasis. However, for publication a few points should be clarified. Find them below.

We thank this reviewer for recognising the importance of our work.

Major points:

-The in cellulo data the KO in the fish point towards a role of DANCR at least in part in the detected phenotype. It would be nice to describe which specific phenotypes are affected by the snoRNAs Vs DANCR. Are the phenotypes affected by DANCR related to melanocyte biology?

Our loss-of-function studies showed that *dancr* F0 crispants exhibit multiple defects in anteroposterior axis development, with the most severely affected embryos lacking defined trunk and tail structures and displaying reduced head size. We also found that *dancr* F0 crispant embryos developed pigmented melanocytes as evidenced at 2dpf (see below). However, the *dancr* crispant embryos at this stage displayed such a severe and variable phenotype along the anterior to posterior axis that it was difficult to quantify the number and location of melanocytes across the embryo, so that an assessment of how *dancr* loss affects melanocyte biology will be more compelling in a defined mutant line. We thus focused our investigation on classifying mutant embryos based on the severity of the observed phenotypic abnormalities as such profound effects on development are unusual for a lncRNA loss-of-function animal model and represent a significant discovery.

We have now performed additional rescue experiments to determine the contribution of *dancr*, *snora26* and *snora27* to the loss-of-function phenotype as requested. Co-injection of *dancr* transcript reduced the proportion of class I embryos with the most severe phenotype (Fig 6C), these embryos lack defined trunk and tail tissue and have a reduced head size. Conversely, our new data show that co-injection of *snora26* and *snora27* in F0 crispants increased the proportion of embryos with severe phenotypic abnormalities (Fig 6D). Whilst we can’t at the moment provide a mechanism behind this, these data strongly suggest that the *dancr* locus expresses a functionally relevant lncRNA and two snoRNA molecules with important roles in early development.

-In Figure 2B, the KD of MITF in SK-MEL-28 is unsuccessful; this is unlikely to be due to the specific cell line, as KD of MITF has been performed successfully by several groups in these line. The experiment should be repeated, possibly with validated siRNAs. Also, it is not clear why these cell lines were chosen, and an explanation for the different outcome of MITF KD in A375 and Mel501 is not provided. In this respect, it would be helpful to compare the relative expression levels of MITF and MYC in the different cell lines, whether CNA or mutations of DANCR exist, and whether they include or not the promoter region.

We used multiple well characterised BRAFV600E melanoma cell lines to make general observations regarding c-MYC and MITF control of *DANCR* expression in melanoma and avoid basing conclusions on studies in a single cell line. These experiments showed that c-MYC activates *DANCR* expression in all cell lines tested, whereas MITF can activate or repress *DANCR* in melanoma in a cell line dependent manner. Whilst we do not know the reason for this, we believe it is important to report these cell type dependent differences.

We use experimentally validated MITF siRNAs used by several different groups (for example [1]) but have always had problems achieving high levels of MITF depletion in SKMEL28 cells). We do not know the basis for this as these siRNAs effectively deplete MITF in 501mel and A375 cells and siRNA transfection is efficient to substantially knock down MYC and DANCR in SKMEL28 cells. This is not due to differences in MITF expression as 501mel and SKMEL28 cells both express a proliferative transcriptional signature characterised by similarly high levels of MITF. We included the SKMEL28 cell data showing that when MITF levels were not depleted no significant change in *DANCR* expression was observed as a control for specificity to reenforce our discovery that *DANCR* is a MITF target in melanoma. Together these data clearly show that changes in MITF lead to changes in *DANCR*.

-Figure 3A: It is not clear whether this is one or more experiments and whether the KD is statistically significant.

Sorry for the confusion. The data in Fig 3A shows mean relative *DANCR* expression in three biological replicates used for the RNA-seq. Statistical analysis using a two-tailed two sample t-test showed that *DANCR* knockdown is significant (p<0.0001). The figure has been updated to display significance and the statistical test is described in the legend.

-Figure 3D: From this picture, it looks like siRNA 2 is the most efficient at knocking down DANCR, is thus not clear the rational for using siRNA 1 for the sequencing.

We observed no significant difference in the efficiency of *DANCR* depletion using either siDANCR1 or siDANCR2 in SKMEL28 cells. Our RNA-seq and RT-qPCR data both show that we effectively depleted *DANCR* expression by 80-85% using siDANCR1 (Fig 3A and Supplemental Table 3).

-Figure 3E-H: To estimate the effect on proliferation and wound healing, also day 1 and 2 should be shown in the proliferation assay. This would also allow proper normalisation of the data in G and H. Knowing that invasion and proliferation in melanoma are mutually exclusive, I would not be surprised to discover that the effect on the closure of the wound is a consequence of the lack of cell proliferation.

We agree that a decrease in proliferation following *DANCR* depletion could lead to reduced wound closure. We therefore specifically addressed this by first treating cells with mitomycin C to inhibit proliferation before seeding in a wound healing assay. This enabled us to isolate the effects on wound closure due to disrupted migration and not because of changes in proliferation. This is described in the Methods section and the Figure 3 legend.

-Mechanistically, the study concludes that DANCR regulates the expression of a good number of transcripts both in human melanoma lines and zebrafish, converging towards regulation of cell death and p53 signalling. Could the authors elaborate on the mechanism? Is this a function of the nuclear portion of the transcript? In that case, is the stoichiometry consistent with the hypothesis?

*DANCR* is highly expressed compared to the majority of lncRNAs and whilst it is cytoplasmically enriched it still contains a significant nuclear portion. As such, it has been reported to function both in the nucleus by recruiting chromatin regulators and/or modulating the activity of transcription factors to directly regulate transcription, as well as post-transcriptionally in the cytoplasm either as a competitive endogenous RNA or to affect mRNA stability and/or translation. Our data are consistent with both nuclear and cytoplasmic mechanisms of action and don’t discriminate between them. Future work will identify *DANCR* interaction partners and define its precise mode of gene regulation.

We have modified the discussion to elaborate on this as suggested.

Minor:

-I detected many typos (e.g. page 9 CRIPSRi) and, especially in the discussion, a lot of references are missing (e.g. page 18 "DANCR knockdown in human organotypic epidermal tissue induced expression of differentiation genes." )

Thanks for drawing this to our attention. We carefully checked the document for typos and missing references and corrected these.

-It is not clear why a zebrafish developmental model was chosen over a zebrafish melanoma model.

Most of what is known about *DANCR* comes from cancer cell line studies. We chose a zebrafish developmental model to enhance our understanding of its role in normal development as nothing is known about this. Moreover, genes involved in development are frequently mis-regulated in cancer.

Reviewer #2: The manuscript by Jones et al., describes the identification of a long non-coding RNA that the authors called DANCR. The DANCR lncRNA is conserved throughout vertebrate evolution at the genomic position, in respect to its protein-coding neighbouring genes, USP46 and RASL11b. DANCR is under the regulation of MITF and c-MYC transcription factors with the established role in melanomagenesis. Through a series of knock-down assays in human melanoma cell lines, the authors propose that DANCR acts in the cytoplasm to control cell proliferation and migration through the regulation of a set of cancer-associated genes. Furthermore, the authors explore the function of the Dancr syntologous transcript in development in distantly related zebrafish. The authors describe a profound effect of Dancr loss of function on early embryonic development suggesting that this lncRNA has an important biological function already in species at the base of vertebrate evolution.

The study has the potential of being of interest to the broad scientific audience. However, several important points below should be addressed.

We appreciate that this reviewer recognised the broad significance of our work.

Major concerns:

1. Phenotypic characterisation of Dancr loss of function in the zebrafish embryos:

Is there a specific reason for restricting analyses of the phenotypic consequences of Dancr loss to CRISPants (zebrafish embryos transiently expressing four guide RNAs targeting the promoter region of Dancr)? Whereas CRISPant analyses gain promising results, they are also prone to artefacts. Simultaneous usage of four different gRNAs also increases the risk of off targeting. A gold standard in the field is generation of stable mutants that should be backcrossed several times to outcross any potential off target mutations. The range of severity of phenotypes achieved in CRISPants strongly suggest that there are some artefactual effects, which should be clarified by generating stable mutants.

Whereas CRISPants offer an advantage of carrying out rescue experiments. However, the observed phenotypes should be confirmed in stable mutants. If confirmed, rescue experiments can be done using CRISPants.

Our loss-of-function analysis of the *dancr* locus using F0 CRISPants has generated significant insights into its role in zebrafish development. This approach was selected due to its rapid turnaround compared the length of time needed to generate stable mutant lines. It enables efficient biallelic knockout in >90% of injected embryos and effectively recapitulates phenotypes observed in established stable lines [2]. We used four guides to improve the efficiency of genetic disruption as previously described [3]. This can result in genetic mosaicism and lead to a range of severity of phenotypes among F0 CRISPants, as reflected in our data.

To evaluate the specificity of the observed loss-of-function phenotype, we therefore performed rescue experiments. These showed that co-injection mature *dancr* IVT RNA significantly reduced the proportion of F0 crispant embryos with the most severe phenotype in a dose dependent manner. This indicates that the abnormal phenotype is not likely to be caused by off-target artefacts but is due to a specific function of the dancr lncRNA.

We agree that the generation of stable mutants is an important next step to further validate our findings. To this end, phenotypically normal dancr F0 CRISPant sibling embryos were raised to adulthood. Adult F0 CRISPants with potential germline transmission of *dancr* loss-of-function mutations were outcrossed to wild-type AB fish to produce F1 progeny. Two females were identified as mutant carriers and each was subsequently outcrossed to wild-type AB fish to generate independent F2 lines.

Randomly selected adult fish from the F2 generation of female 1 were crossed with those from the F2 generation of female 2. The progeny from four pairs were screened for phenotypic abnormalities. In one pair, 57 embryos exhibited phenotypes consistent with the original F0 CRISPant loss-of-function phenotype (see below), 14 embryos failed to develop, and 161 developed normally. The progeny from the remaining three pairs developed normally. This clearly shows the CRISPant phenotypes observed are identical to the stable mutant phenotypes and suggests that there are very little, if any, artefactual effects of using four gRNAs.

The generation of a stable transgenic line is predicted to take a further 3-4 months and is beyond the scope of the current manuscript. The line will need to be maintained in the form of healthy heterozygous animals. To establish this, the pair that produced embryos with phenotypes will need to be sequenced to determine whether they carry the same mutation. Genotyping also needs to be done for the F3 embryos to find out whether the ones displaying phenotypes are homozygous for the mutation. Once these are confirmed, the healthy offspring need be raised to adulthood (3 months from now) to establish the F3 generation. Genotyping will need to be done again to identify heterozygous carriers. Once this pool of heterozygous animals becomes available, rescue experiments can be performed.

2. The connection between human melanoma and zebrafish embryonic development: Without making a clear link between the two phenomenon, melanomagenesis and early embryonic development, the logic of the manuscript is not clear. The authors state that the human and zebrafish lncRNAs potentially regulate same genes. However, there is no direct evidence for it. One way to connect zebrafish development and human melanoma is to perform rescue experiments with the human DANCR transcript. It appears that the developmental effects observed in zebrafish are much more pronounced that the human effect of DANCR. This again suggests that the phenotype of transient CRISPants should be confirmed/clarified in stable mutants.

As suggested, we co-injected human *DANCR* lncRNA with the zebrafish *dancr* promoter targeting Cas9-crRNA-tracrRNP complex in a genome editing experiment to test for functional conservation. Human *DANCR* increased the proportion of F0 crispant embryos with the most severe phenotypic abnormalities (characterised by a lack of defined trunk and tail tissue and a reduced head size). This suggests that human *DANCR* can modify normal development in zebrafish and that aspects of its developmental function may be conserved.

Whilst the developmental effects observed in zebrafish appear more pronounced than the effect of *DANCR* in melanoma, its wider role in human embryonic development beyond a proposed role in the suppression of progenitor cell differentiation has not been studied. Most of what is known about human *DANCR* is from studies in cancer. These showed that *DANCR* expression is not restricted to melanoma and is co-expressed with essential growth promoting genes across many different types of cancer, suggesting that human *DANCR* has a wide range of essential functions in multiple cell types [4, 5]. This is consistent with the broad expression pattern observed for *dancr* in the developing zebrafish embryo and the wide spectrum of phenotypic abnormalities observed in *dancr* F0 crispant embryos.

Additional specific comments:

3. Deletion of Dancr promoter region in zebrafish embryos is an important point that should be more elaborated in the manuscript. What is the expected size of the promoter region deletion? Figure 5A should show position of primers used in Figure 5B for qRT-PCR.

We determined the size of the genomic deletions in four random phenotypically abnormal crispant embryos using PCR and validated *dancr* promoter deletion using Sanger sequencing. Alignment to the zebrafish genome shows that crispant embryos contain specific deletions of varying sizes compared to wild type sequence and that the deletions map to the locations of the sgRNAs used. This was expected due to the use of multiple sgRNAs and is described in Supplemental Figure 3B, C. We have made the reference to this figure clearer in the main text and have added RT-qPCR primer locations onto Figure 5A as requested.

4. A clearer schematic presentation of genome editing strategy and genotyping would be very useful. The authors should provide also rationale for the usage of four gRNAs and not two gRNAs which are more standard.

As described above, the locations of the sgRNAs used in the genome editing strategy and representative validation of genomic deletions are described in Supplemental Figure 3B, C.

We found that two guides generated mutations with low efficiency and followed the guidance in [3] to use four guides to improve this. This work reported that targeting a single gene using four guide RNAs increases editing efficiency to up to 98% whilst consistently reproducing germline-transmitted knockout phenotypes.

5. In Figure 6A, the authors show a range of phenotypes achieved when embryos are transiently express four gRNAs targeting Dancr promoter. For the RNA-seq analysis on CRISPants (Figure 5C), embryos with which grade of phenotypic abnormalities were used? How many biological replicates were used for RNA-seq. The authors should show also PCA analyses.

We apologize for the confusion. RNA-seq was performed using class I and II F0 dancr embryos and three biological replicates were used. We have updated the transcriptomics methods to include this information.

PCA analysis of the RNA-seq reads was performed as requested and the results are displayed in Supplemental Fig1A (human) and Supplemental Fig 3D (zebrafish). These showed that the control and knockdown samples cluster separately in both cases.

6. Figure 3B: were GO term analyses done on both, significantly up-regulated and down-regulated genes? This should be indicated.

GO analysis was performed on all de genes. We have modified the results text to make this clearer.

7. “We next investigated the function of the zebrafish orthologue of DANCR in development”: Those are not exactly orthologs but syntologs or positional orthologs. The authors should be clear about their nomenclature.

This is a good point and we agree that lncRNA nomenclature across species still remains confusing, especially given the debate over the extent of lncRNA functional conservation. We now use the terms “positional orthologue” or “positionally equivalent” instead of “orthologue” throughout the manuscript.

A detailed sequence comparison should be carried out to see if promoter region or some of the splice junctions are conserved between distantly related DANCR transcripts.

As requested, we examined the human and the zebrafish locus using comparative genomics data from the UCSC browser. The sequences of the splice sites of *DANCR* and regions in its promoter are conserved throughout mammals, but genome alignment does not extend beyond to other vertebrates. When examining the zebrafish locus, alignment to other teleost fish species, that are further apart from each other compared to mammals, shows no alignability beyond the snoRNA loci. We note that splice sites and transcription factor binding sites are too short to support alignability of lncRNA loci across large evolutionary distances. These results are shown in a new Supplemental Figure 3A.

8. The authors should do a sub-cellular fractionation experiment also in zebrafish, not only in cell lines to conclude on Dancr subcellular localization in zebrafish. The single molecule in situ hybridisation has rather low resolution to fully support cytoplasmic localization of Dancr.

The fractionation experiment to determine the sub-cellular localisation of zebrafish *dancr* (Fig 1E) was performed using whole zebrafish embryos at 30 hpf as indicated in the methods.

9. As the study does not provide any molecular insides into the mode of action of DANCR, sentences such as “…human and zebrafish DANCR may exert similar functions or work using similar mechanisms of action” should be removed or re-phrased.

This sentence states our working hypothesis describing the proposed molecular basis for *DANCR* functional conservation. It does not explicitly state that human and zebrafish *DANCR* work in the same way, and we take care to use the words “suggest” and “may” when proposing hypotheses. *DANCR* mode of action will be investigated in future work.

10. Is zebrafish Dancr also under control of MITF and c-MYC?

To address this, we first examined the genomic sequence encompassing the zebrafish *dancr* locus using the JASPAR database of transcription factor binding motifs. This identified a MITF consensus motif <1kb downstream of the locus (Supplemental Figure 3A). To determine if MITF disruption leads to changes in *dancr*, we measured *dancr* expression in homozygous nacre mutant whole zebrafish embryos. These fish contain a loss-of-function mutation in the zebrafish *mitfa* gene and homozygous mutants display a complete loss of melanocytes. RT-qPCR analysis revealed a significant reduction in *danc*r expression in *mitfa^-/-^* fish compared to wild type controls, indicating that *dancr* is transcriptionally regulated by MITF in zebrafish. This finding is supported by our RNAscope data, which show co-expression of *mitfa* and *dancr* in presumptive melanoblasts (Fig 4E).

Reviewer #3: This paper from Jones and collaborators aims to clarify the role of the DANCR lncRNA in melanoma and during zebrafish embryogenesis. The pro-tumorigenic role of DANCR has been demonstrated in several cancer types and some of the regulated pathways have been investigated by other works. However, the role of DANCR in melanoma has been poorly investigated and no data are available on zebrafish development. Therefore, the presented data are novel and interesting, although mechanistic characterization of the DANCR role at molecular level is superficial. The experiments are generally well conducted with relevant controls in place and the paper is well-written, with a clear rationale.

We thank this reviewer for acknowledging that our data are novel and interesting, the experiments are well conducted and the paper is well written.

However, I have some specific issues that may be addressed before publication:

1. The Authors showed that DANCR is mainly localized in the cytoplasm, even if a small fraction is retained into the nucleus. Then, they used RNA-sequencing to characterize the consequences of DANCR knockdown in both melanoma cell line and zebrafish model. In my opinion, this approach would be more suitable for lncRNA having nuclear localization and putative transcription-regulating function. Indeed, they found only a small number of transcripts deregulated. The possible function(s) of DANCR in the nucleus may be better discussed to justify this choice and to further highlight the complexity of this lncRNA activities.

We respectfully disagree that our experimental approach is more suitable for investigating a nuclear localised lncRNA. It is well documented that cytoplasmically enriched lncRNAs are most effectively suppressed using siRNAs, see [6] for a representative study including *DANCR*, and that knockdown of a cytoplasmic lncRNA can lead to large-scale changes in gene expression.

We report in the response to reviewer #1 that *DANCR* can function both in the nucleus to directly regulate transcription as well as post-transcriptionally in the cytoplasm either as a competitive endogenous RNA or to affect mRNA stability and/or translation. We have modified the manuscript to include a discussion on possible nuclear and cytoplasmic DANCR functions as suggested.

2. The extent of overlap between genes deregulated upon DANCR knockdown in melanoma cell line and in zebrafish may be clearly represented in Figure 5. They mentioned these genes in the discussion section, but they should be depicted somehow also in the Figure.

We did not generate a figure to illustrate the extent of overlap as this only identified a single gene, *Cdkn1a*, and thought this was best represented in the text. The discussion highlights the fact that we also discovered a number of related genes in similar families that are regulated by both human and zebrafish *DANCR*.

3. Validation by qRT-PCR of at least some of the deregulated genes is required (Figure 3 and Figure 5). A validation of genes deregulated in SK-MEL-28 also in a second melanoma cell line would add robustness to the entire work.

To further validate the specificity of our gene expression experiments and add robustness as suggested, we depleted *DANCR* expression in A375 melanoma cells using siDANCR1 and siDANCR2 and performed RT-qPCR to measure changes in the expression of four key *DANCR* target genes (*CDKN1A*, *CCND1*, *DKK1*, *PTK2*). *DANCR* knockdown led to a reduction in the expression of all four genes with both siRNAs (Supplemental Figure 1B), consistent with the SK-MEL-28 RNA-seq data.

4. In Figure 6, the Authors show that CRISPR-mediated deletion of the DANCR promoter region induces defects in zebrafish embryogenesis. These defects are partially rescued by re-expressing the DANCR mature transcript. However, the Authors could not rule out the possible contribution of snora26 or snora27 elimination to the phenotype. Is it possible that deleting the DANCR promoter, the chromatin status of the entire genomic locus has been altered? In other words, is the expression of the neighbor genes, such as usp46 or rasl11b, altered upon DANCR promoter deletion?

We have now performed rescue experiments to assess the possible contribution of *snora26* and *snora27* to the *dancr* loss-of-function phenotype. Instead of restoring normal function, co-injection of *snora26* and *snora27* in F0 crispants increased the proportion of class I embryos with severe phenotypic abnormalities. These data are shown in a new Fig 6D. Although we don’t understand the mechanism behind this, the results suggest that *snora26* and *snora27* are biological relevant transcripts that can contribute to the developmental function of the *dancr* locus.

No significant changes in expression of the neighbouring *usp46* or *rasl11b* genes were detected in our RNA-seq analysis of F0 crispant embryos (DESeq2 padj<=0.05; log2FoldChange>=1.0). This indicates that the chromatin status of the whole locus has not been altered by the short *dancr* promoter deletion.

**Have all data underlying the figures and results presented in the manuscript been provided?**
Large-scale datasets should be made available via a public repository as described in the *PLOS Genetics* [data availability policy](https://eur01.safelinks.protection.outlook.com/?url=http%3A%2F%2Ftrack.editorialmanager.com%2FCL0%2Fhttp%3A%252F%252Fjournals.plos.org%252Fplosgenetics%252Fs%252Fdata-availability%2F1%2F010f019812faa1ca-7572be3c-dc5b-42ac-a485-67b8c370ada6-000000%2F0xxqTVl7t0jowwhyNAxTAFzSLi3HEV80Eu3pzfSYPcI%3D219&data=05%7C02%7Ckwv20%40bath.ac.uk%7C3339e12e4ed648b4d05208ddc45b56ef%7C377e3d224ea1422db0ad8fcc89406b9e%7C0%7C0%7C638882618765086921%7CUnknown%7CTWFpbGZsb3d8eyJFbXB0eU1hcGkiOnRydWUsIlYiOiIwLjAuMDAwMCIsIlAiOiJXaW4zMiIsIkFOIjoiTWFpbCIsIldUIjoyfQ%3D%3D%7C0%7C%7C%7C&sdata=IN7iu%2BbEjeUQ4hDrfjCXO0q9rpkmivdE5k1llf5oyYw%3D&reserved=0), and numerical data that underlies graphs or summary statistics should be provided in spreadsheet form as supporting information.

Reviewer #1: Yes

Reviewer #2: Yes

Reviewer #3: **No:** Numerical data underlying the graphs in the Figures have not been provided.

The numerical data underlying the graphs are displayed in a new Supplemental Table 6.

PLOS authors have the option to publish the peer review history of their article ([what does this mean?](https://eur01.safelinks.protection.outlook.com/?url=https%3A%2F%2Ftrack.editorialmanager.com%2FCL0%2Fhttps%3A%252F%252Fjournals.plos.org%252Fplosgenetics%252Fs%252Feditorial-and-peer-review-process%2523loc-peer-review-history%2F1%2F010f019812faa1ca-7572be3c-dc5b-42ac-a485-67b8c370ada6-000000%2F4wMHFfmifL_lWngu2QLMEtkwCeRJzmADShG1dnO-TiI%3D219&data=05%7C02%7Ckwv20%40bath.ac.uk%7C3339e12e4ed648b4d05208ddc45b56ef%7C377e3d224ea1422db0ad8fcc89406b9e%7C0%7C0%7C638882618765121835%7CUnknown%7CTWFpbGZsb3d8eyJFbXB0eU1hcGkiOnRydWUsIlYiOiIwLjAuMDAwMCIsIlAiOiJXaW4zMiIsIkFOIjoiTWFpbCIsIldUIjoyfQ%3D%3D%7C0%7C%7C%7C&sdata=XsVb3pb3CEwX1LHLLNsEPfx%2B15E9ymQo%2FYO8VdHabpU%3D&reserved=0)). If published, this will include your full peer review and any attached files.

If you choose “no”, your identity will remain anonymous but your review may still be made public.

**Do you want your identity to be public for this peer review?** For information about this choice, including consent withdrawal, please see our [Privacy Policy](https://eur01.safelinks.protection.outlook.com/?url=https%3A%2F%2Ftrack.editorialmanager.com%2FCL0%2Fhttps%3A%252F%252Fwww.plos.org%252Fprivacy-policy%2F1%2F010f019812faa1ca-7572be3c-dc5b-42ac-a485-67b8c370ada6-000000%2FNDiYzQ5JmWuBGFRNLqwbb0iYHloAEKBGOS9FKMjWG-g%3D219&data=05%7C02%7Ckwv20%40bath.ac.uk%7C3339e12e4ed648b4d05208ddc45b56ef%7C377e3d224ea1422db0ad8fcc89406b9e%7C0%7C0%7C638882618765133135%7CUnknown%7CTWFpbGZsb3d8eyJFbXB0eU1hcGkiOnRydWUsIlYiOiIwLjAuMDAwMCIsIlAiOiJXaW4zMiIsIkFOIjoiTWFpbCIsIldUIjoyfQ%3D%3D%7C0%7C%7C%7C&sdata=Cf7orm294GY1PMji3PUKXEWfb2YAPOZEEHz%2Bvw%2FxSAE%3D&reserved=0).

Reviewer #1: No

Reviewer #2: No

Reviewer #3: No

[NOTE: If reviewer comments were submitted as an attachment file, they will be attached to this email and accessible via the submission site. Please log into your account, locate the manuscript record, and check for the action link "View Attachments". If this link does not appear, there are no attachment files.]

**Figure resubmission:**

While revising your submission, please upload your figure files to the Preflight Analysis and Conversion Engine (PACE) digital diagnostic tool, <https://pacev2.apexcovantage.com/>. PACE helps ensure that figures meet PLOS requirements. To use PACE, you must first register as a user. Registration is free. Then, login and navigate to the UPLOAD tab, where you will find detailed instructions on how to use the tool. If you encounter any issues or have any questions when using PACE, please email PLOS at [figures@plos.org](mailto:figures@plos.org). Please note that Supporting Information files do not need this step. If there are other versions of figure files still present in your submission file inventory at resubmission, please replace them with the PACE-processed versions.

**Reproducibility:**

To enhance the reproducibility of your results, we recommend that authors of applicable studies deposit laboratory protocols in protocols.io, where a protocol can be assigned its own identifier (DOI) such that it can be cited independently in the future. Additionally, PLOS ONE offers an option to publish peer-reviewed clinical study protocols. Read more information on sharing protocols at <https://plos.org/protocols?utm_medium=editorial-email&utm_source=authorletters&utm_campaign=protocols>

*In compliance with data protection regulations, you may request*

1.	Carreira S, Goodall J, Denat L, Rodriguez M, Nuciforo P, Hoek KS, et al. Mitf regulation of Dia1 controls melanoma proliferation and invasiveness. Genes & development. 2006;20(24):3426-39. doi: 10.1101/gad.406406. PubMed PMID: 17182868; PubMed Central PMCID: PMC1698449.

2.	Kroll F, Powell GT, Ghosh M, Gestri G, Antinucci P, Hearn TJ, et al. A simple and effective F0 knockout method for rapid screening of behaviour and other complex phenotypes. eLife. 2021;10. Epub 20210108. doi: 10.7554/eLife.59683. PubMed PMID: 33416493; PubMed Central PMCID: PMCPMC7793621.

3.	Wu RS, Lam, II, Clay H, Duong DN, Deo RC, Coughlin SR. A Rapid Method for Directed Gene Knockout for Screening in G0 Zebrafish. Dev Cell. 2018;46(1):112-25 e4. doi: 10.1016/j.devcel.2018.06.003. PubMed PMID: 29974860.

4.	Lu Y, Hu Z, Mangala LS, Stine ZE, Hu X, Jiang D, et al. MYC Targeted Long Noncoding RNA DANCR Promotes Cancer in Part by Reducing p21 Levels. Cancer research. 2018;78(1):64-74. Epub 2017/11/29. doi: 10.1158/0008-5472.CAN-17-0815. PubMed PMID: 29180471; PubMed Central PMCID: PMCPMC5993051.

5.	Mitra R, Adams CM, Eischen CM. Systematic lncRNA mapping to genome-wide co-essential modules uncovers cancer dependency on uncharacterized lncRNAs. eLife. 2022;11. Epub 2022/06/14. doi: 10.7554/eLife.77357. PubMed PMID: 35695878; PubMed Central PMCID: PMCPMC9191893.

6.	Lennox KA, Behlke MA. Cellular localization of long non-coding RNAs affects silencing by RNAi more than by antisense oligonucleotides. Nucleic Acids Res. 2016;44(2):863-77. Epub 2015/11/19. doi: 10.1093/nar/gkv1206. PubMed PMID: 26578588; PubMed Central PMCID: PMCPMC4737147.
